# Assessing cancer in people with profound and multiple disabilities

**DOI:** 10.1186/s12885-023-11313-3

**Published:** 2023-08-25

**Authors:** Daniel Satgé, Motoi Nishi, Brigitte Trétarre

**Affiliations:** 1 Oncodéfi, 209 Avenue des Apothicaires, Parc Euromédecine, Montpellier, 34090 France; 2 UMR 1302 Institute Desbrest of Epidemiology and Public Health, INSERM, Univ Montpellier, Montpellier, France; 3 Department of Fundamental Health Sciences, Health Sciences University of Hokkaido, Tobetsu, Japan; 4 Registre des Cancers de l’Hérault, 208 Avenue des Apothicaires, Montpellier, 34090 France; 5 Center for Epidemiology and Research in Population Health (CERPOP), Toulouse, France

**Keywords:** Cancer, Cancer incidence, Cancer screening, Intellectual disability, Profound and multiple disabilities

## Abstract

**Background:**

Cancers are as common in individuals with intellectual disabilities as in the general population (GP). For the subgroup of people with profound and multiple disabilities (PMD) who present with both severe intellectual disability and major motor disorders, the frequency and distribution of cancers are currently not known, preventing proper cancer surveillance.

**Methods:**

We carried out a systematic and synthetic review of the medical literature, including a focused search of Japanese data.

**Results:**

The total risk of cancer in individuals with PMD is thought to be lower than in the GP, possibly due to a shorter life expectancy. They have reduced exposure to cancer risk factors, such as alcohol, tobacco, sunlight, human papillomavirus infection, occupational toxins, and being overweight. On the other hand, individuals with PMD present a greater frequency of gastroesophageal reflux disease, *Helicobacter pylori* gastritis, chronic cystitis, and cryptorchidism, which increase the risk for cancer of the esophagus, stomach, urinary bladder, and testes. In addition, certain genetic disorders underlying compromised motor and cognitive functions are associated with higher risk of childhood cancers. An analysis of 135 cancers in persons with PMD in Japan suggested that they present a particular tumor profile, with certain cancers rarer than in the GP, whereas cancers of the digestive tract are frequent. Cancers of the digestive tract occurred significantly earlier than in the GP (colon: average age 48.3 years vs. 71.3 years in the GP, esophagus: 39 years vs. 72 years in the GP). An increasing number of therapeutic successes in children and adults with PMD have been reported in different countries when cancers are discovered early.

**Conclusion:**

Individuals with PMD must be appropriately monitored for cancer. Screenings for breast and colon cancer, as well as regular monitoring of the esophagus, stomach, urinary bladder, and testicles, are necessary. Population-based epidemiological studies are needed to better understand risk factors, frequency, and distribution of cancers in the PMD population.

**Supplementary Information:**

The online version contains supplementary material available at 10.1186/s12885-023-11313-3.

## Background

Intellectual disability (ID) is characterized by cognitive dysfunction associated with adaptive deficits, regardless of their origin, appearing at birth or through 18 years of age. Individuals with ID represent 1–2% of the population and develop cancer as commonly as individuals in the general population [1, 2]. A subset of people with ID have profound and multiple disabilities (PMD) (or polyhandicap), a combination of severe or profound ID and severe motor impairment [3]. Though cancer occurrence has been studied among the whole ID population, little data exists specific to people with PMD. Current information on cancer pathology is based on knowledge about cancers in individuals with severe and profound intellectual disabilities (SPID) and individuals with cerebral palsy (CP). Additional studies are needed to understand cancer occurrence among individuals with PMD and to better identify risk factors and inform surveillance strategies for this population.

Individuals with PMD share many common features with the larger group of people with ID in terms of cancer risk factors and organ vulnerability. However, differences are more marked between individuals who have a mild impairment and those with a moderate impairment. Furthermore, the characteristics of individuals with PMD—a vulnerable population that is dependent on caregivers—necessitates adapted medical follow-up. This review synthesizes, for the first time, available knowledge on cancer in a recently isolated and poorly understood group.

## Methods

A literature search for relevant articles was conducted using PubMed and Google Scholar without specification for language or publication date. The following search terms were used: “profound and multiple disabilities, OR profound intellectual and multiple disabilities, OR profound and complex disabilities, OR severe intellectual and motor disabilities, OR severe multiple disabilities, OR complex intellectual and sensory disabilities, OR children with complex needs”, which were crossed with “cancer OR neoplasms”. A complementary manual search was conducted with the words “profound and multiple disabilities” AND separately “malignancy”, “solid tumors”, “neoplasia”, “sarcoma”, “carcinoma”, “adenocarcinoma”, “leukemia”, “lymphoma”, “neuroblastoma”, “hepatoblastoma”, “brain tumors”, “myeloma”, and “melanoma”. Studies providing data on the frequency of cancers for individuals with SPID did not specify whether ID was associated with motor co-morbidities; therefore, we could not precisely delineate those individuals with PMD. Thus, research was performed by supplementing the scarce data on PMD with available information on cancer in individuals with SPID and data on individuals with marked motor disorders. We cross-searched the following key words: “severe profound intellectual disability” and “cerebral palsy” with “malignancy”, “solid tumors”, “neoplasia”, “sarcoma”, “carcinoma”, “adenocarcinoma”, “leukemia”, “lymphoma”, “neuroblastoma”, “hepatoblastoma”, “brain tumors”, “myeloma”, and “melanoma”. A manual search was also performed using references collected by the team on cancer and ID for previous research.

The search yielded 363 articles. First, we assessed the titles and abstracts of all search results, revealing 83 duplicate articles. Among the 280 non-duplicate articles, titles and abstracts were scanned, and then reference lists were scanned. This step retained 93 full articles and conference communications with potentially eligible studies. After the research team read these 93 articles, 53 articles were retained for inclusion.

**Fig. 1:**
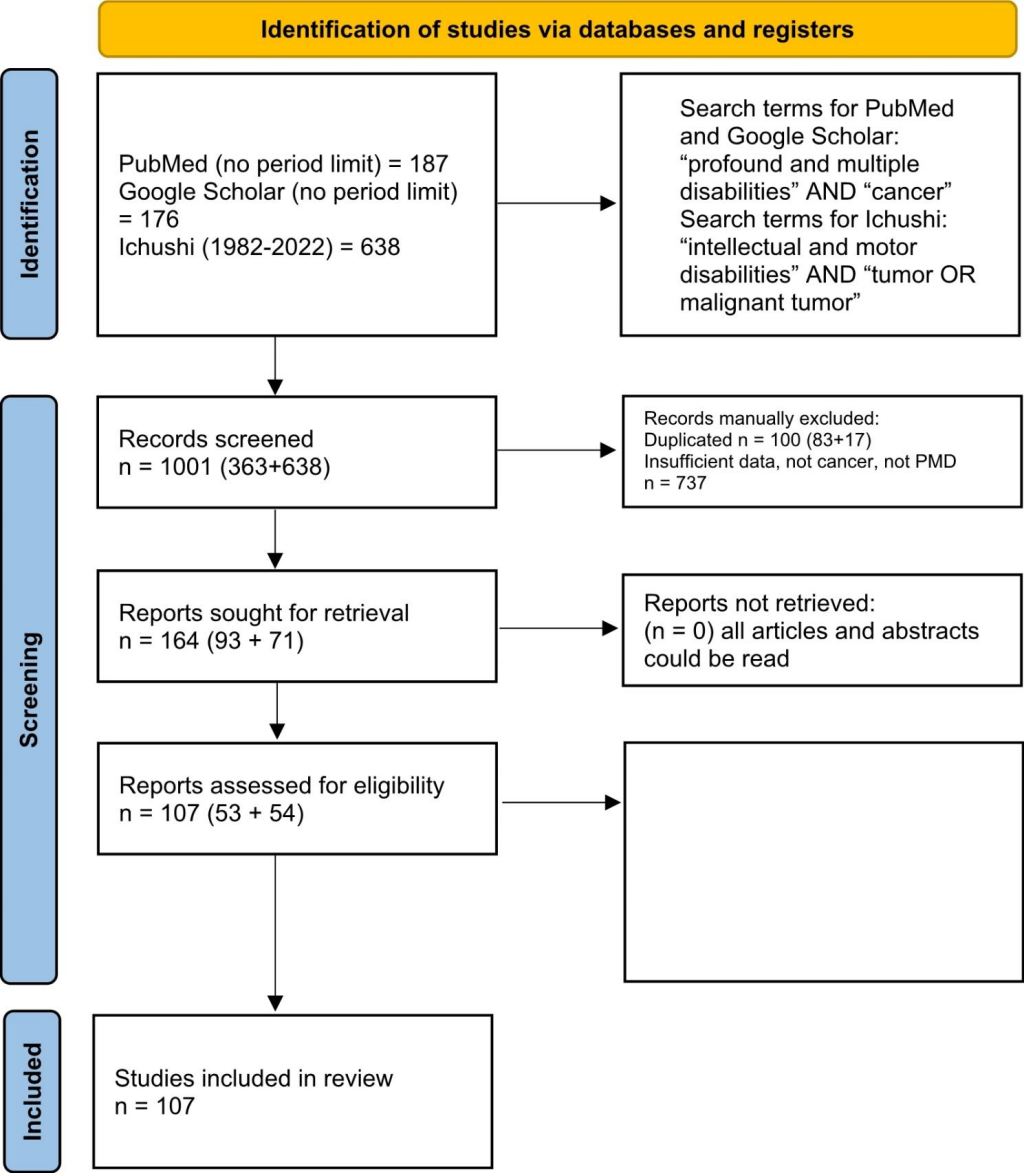
Flow chart: Cancer in people with profound and multiple disabilities

One researcher (MN) noticed that the medical literature printed in Japanese contained articles on cancer in individuals with PMD. Thus, an additional search was conducted of Japanese publication sources using “Japanese Medical Abstracts” (ICHUSHI) data from 1982 to 2022, which includes medical conference reports. Search terms were “intellectual and motor disability” crossed with “tumor OR malignant tumor”. Given the paucity of the medical literature on cancer in individuals with PMD, conference reports were included in the search. This search yielded 638 results, from which 71 articles and reports were selected after removing 17 duplicates. All Japanese cases were read by one author (MN) and relevant information translated into English; 54 articles and conference reports were included in the review (Fig. 1). Fifteen Japanese articles are cited in the reference list, and the remaining Japanese publications, including five duplicate and one set of triplicate reports, are listed in Supplement A.

## Results

### Incidence and mortality of cancers in people with PMD, SPID, and CP

Table 1 summarizes the studies in the literature providing data on cancer incidence and cancer mortality in people with PMD, SPD, SPID, or CP. The table shows that the four articles on cancers in PMD are not population-based. In contrast, six of eight publications in national and regional cancer registries in Europe and the USA on cancers in SPID and CP are population-based but provide indirect information. Though much of the global medical literature does not distinguish PMD as a distinct subset of ID, the Japanese literature reports data on cancer specific to this subgroup. One article was published on the subject before 2000 [4]; other reports were printed after policy modifications in 1999 [5]. In 2009, approximately 40,000 persons among Japan’s 127,000,000 inhabitants were estimated to have PMD. Among those with PMD, 23,000 lived with their family, 10,000 were residents of institutions dedicated to persons with PMD, and 7,000 were in long-stay residential hospitals [5].

**Table 1:** Cancer frequency reported in the medical literature for patients with profound and multiple disability, severe and profound disability, or cerebral palsy

Country, author, publication date	Covered period	Sources	Population-based	Cancer frequency
**Profound and multiple disability**				
Japan, Ishizaki, 1990	1970–1990	Medical center, Fuchu (Tokyo)	No	3% (3/98) of PMD patient deaths due to cancer
Japan, Origuchi, 2002	1982–1999	PMD deaths in Japanese hospitals	No	1.6% (37/2354) of PMD patient deaths due to cancer
Japan, Chiba, 2005	NI	Rehabilitation center, Kanagawa prefecture	No	3.5% (6/173) of patients with PMD developed cancer
Japan, Sado, 2020	2009–2018	Medical center Matsumoto, Nagano prefecture	No	5.6% (5/89) of PMD patients developed cancer
**Severe and profound intellectual disability**				
USA, Cleland, 1971	54 years	Austin State School (Texas)	No	0.6% (4/660) of SPID patient deaths due to cancer
USA, Achterberg, 1978	1973–1976	Texas Department of Mental Health	No	3% of SPID patient deaths and 18% of deaths in the general population due to cancer
Denmark, Dupont, 1990	1976–1984	Danish National Service of Mentally Retarded register	Yes	SMR for cancer in males with SPID 0.84, females with SPID 1.35 (p < 0.05)
Finland, Patja, 2001	1962–1997	Finnish national registration of people with ID	Yes	4% of SPID patient deaths (half that of the general population)
UK, Kiani, 2010	1997–2006	Leicestershire Learning Disability register	Yes	SMR 0.94 for people with moderate to severe ID
**Cerebral palsy**				
USA, Day, 2008	1988–2002	California Developmental Services	Yes	Excess mortality for all cancers (SMR 1.31, p < 0.05) and for esophageal (SMR 5.40), colon (2.16), liver (2.21), breast (1.83), urinary bladder (4.57) cancers Decreased SMR for lung cancer (0.22)
France, Duruflé-Tapin, 2014	2000–2008	INSERM registration of death certificates, whole country	Yes	Decreased mortality from cancer in persons with CP: 7% of all deaths compared to 29% in the general population
UK, Ryan, 2019	1998–2015	Clinical research practice data link	Yes	SMR 1.42, not different from the general population (n.s.)

PMD = profound and multiple disability.

SPID = severe and profound intellectual disability.

CP = cerebral palsy.

SMR = standardized mortality ratio.

ID = intellectual disability.

NI = not indicated.

n.s. = not significant.

During 1982–1999, there were 2,354 deaths of hospitalized persons with PMD in Japan, including 37 (1.6%) who had cancer [6]. In a Kanagawa prefecture rehabilitation center, 6 of 173 (3.5%) patients with PMD (aged 20–77 years, mean age 31.3 years) developed a malignant neoplasm [7]. During 1970–1990, 3 of 98 (3%) deaths of persons with PMD at the Fuchu Metropolitan Medical Center (Tokyo) were attributed to cancer [4]. More recently, among 89 patients admitted at the Matsumoto Medical Center in Nagano Prefecture, 5 (6%) had cancer [8]. Importantly, these data are not population-based, but summarize hospital statistics. Therefore, estimating cancer incidence and mortality in the subpopulation with PMD may be facilitated by assessing data from individuals with SPID and CP.

Older studies considered individuals with SPID to be protected against or less frequently affected by cancer. An institution in Texas (USA) reported that only 4 of 660 (0.6%) deaths of individuals with profound mental deficit were linked to cancer [9]. Furthermore, a survey conducted in Texas during 1973–1976, which included 857 people, indicated that cancer deaths were six-times less frequent in people with ID (3% of deaths) than in the general population (18% of deaths), and that lower IQ correlated with lower cancer mortality risk [10]. Notably, these results are biased because life expectancy was sharply reduced for persons with SPID, who at that time did not reach the age at which the majority of cancers are observed in the general population. The average age of residents at death was 19.8 years in this study, whereas peak cancer incidence currently occurs in France at 64 years for women and 67 years for men [11].

Since the 1970s, the life expectancy of the subpopulation with SPID has increased due to advances in medicine and improved support. This increase translates to an equivalent risk of developing cancer for individuals with SPID as the general population. A Danish study covering the period 1976–1984 reported a similar number of cancers for 9,891 people with SPID compared to the general population. The standardized mortality ratio (SMR) was little different for men [95% confidence interval (CI) 0.65–1.08] but significantly higher for women (1.35; 95% CI 1.08–1.68) [12]. Similarly, a study of people with SPID in Finland during 1967–1997 found an equivalent incidence of cancer for people with moderate ID (12% of the group) and people with profound disabilities (15% of the group) [1]. The same researchers performed a mortality study indicating a lower risk of death from cancer in individuals with SPID. Persons with a more pronounced deficiency had the lowest rate of cancer death, with 4% of cancer deaths for severe ID, and 11% and 16% for moderate and mild ID, respectively [13]. Another report that combined moderate impairment with severe and profound impairment found equivalent cancer mortality [14].

Among adults with CP, cancer deaths are estimated to be more frequent than in the general population in the United Kingdom [15, 16], but less frequent than in the general population in France [17]. Studies conducted in Europe may be biased because they do not directly compare specific causes of mortality and because of the small sizes of the groups studied compared to a study in the USA [18]. CP increases the frequency of cryptorchidism - a risk factor for testicular cancer - especially when the patient experiences quadriplegia and comorbidities, including SPID [19]. In addition, women with CP are estimated to have double the risk of dying from breast cancer [20]. As individuals with PMD represent only a small proportion (~ 20%) of those with CP, data for individuals with CP should be interpreted with caution.

### Distribution and age at diagnosis of cancers in people with PMD and ID

We reviewed 54 articles and conference communications (listed in Supplement A) from the Japanese medical literature, published predominantly during 2000–2022, with one dated 1990 [4]. These reports noted 135 malignant tumors in 133 patients with PMD (Table 2). Among these tumors, 80 (59%) were of the digestive tract, 37 (27%) the colon, 13 (10%) the esophagus, 11 (8%) the urinary bladder, 8 (6%) the breast, and 5 the lungs (4%). The low frequency of lung and ear, nose, and throat cancers differs from that observed in the general population. Only one case of uterine cervical cancer was reported, a clear cell carcinoma unrelated to human papillomavirus (HPV) infection. The literature also indicates that cancers are frequently diagnosed at an advanced stage among persons with PMD [21–23]. On the other hand, three teams reported cancer discovered early through screening and treated successfully in patients with PMD [24–26]. In addition, age at cancer diagnosis was lower among persons with PMD than in the general population, despite the propensity for diagnosis occurring at advanced stages of disease. For the two most frequent cancers for which age was reported, we calculated a mean age at diagnosis of 39.1 years for esophageal cancer (n = 13) and 48.3 years for colon cancer (n = 25). In the Japanese general population, the mean age at diagnosis for esophageal cancer is 71.3 years and for colon cancer 72 years [27]. This supports Hashimoto and Kodama, who raised the possibility of a younger age of onset for digestive tract cancer [28].

**Table 2:** Summary of 135 malignancies in 133 patients with PMD reported in the Japanese literature (see Supplement A) up to 14 October 2022

Cancer type	Number
**Central nervous system** Brain	**5** 5
**Digestive tract** Esophagus Stomach Colon Liver Appendix Gallbladder Pancreas Gastrointestinal NOS	**80** 13 8^a^ 37^c^ 6^b^ 1 3 1 12
**Urinary tract** Kidney Urinary bladder	**12** 1 11^c^
**Female reproductive system** Breast Uterine cervix Endometrium Ovary	**11** 8 1^d^ 1 1
**Respiratory tract** Frontal sinus Lung	**7** 2 5^c^
**Hematopoietic system** Myelodysplastic syndrome Leukemia Lymphoma Multiple myeloma	**7** 1 4 1^a^ 1
**Other** Testis Thyroid Sarcoma Pelvis Skin Tongue	**13** 5 3 2 1 1 1^e^

Only 3 cases (gastric lymphoma, liver cancer, and colon cancer) were reported before 2000 [4]

NOS = not otherwise specified

^a^ A gastric lymphoma is counted as stomach cancer but not as lymphoma

^b^ Three cases were found in infants

^c^ A patient had both urinary tract cancer and lung cancer; another had colon cancer and urinary bladder cancer

^d^ Clear cell carcinoma in a 15-year-old girl

^e^ In situ carcinoma

Data on the SPID group from the literature in other countries, in which individuals with PMD cannot be isolated, present a different distribution of cancers for both the general population and individuals with mild and moderate ID (Table 3). Those with SPID have greater risk of digestive, testicular, gallbladder, and brain tumors.

**Table 3:** Differences in the incidence of certain cancers according to the level of intellectual disability (ID)

Affected organ	SPID (30 patients)	**Moderate and mild ID** **(87 patients)**
Digestive tract	1.4	1.1
Esophagus	2.5	2
Gallbladder	10.3	1.4
Lung	0.0	0.8
Prostate	0.4	0.2
Testis	9.9	2.1
Urinary tract	0.9	0.2
Central nervous system	3.5	0.6
Thyroid	3.1	2.1
Lymphoma	1.2	1.5

Values are presented as standardized incidence ratio (SIR) compared to the general population. For example, for people with severe to profound ID, gallbladder cancers have an SIR of 10.3 compared to the general population, whereas the SIR is only 1.4-fold higher for people with mild and moderate ID compared to the general population. Data are not provided for female reproductive organs. SPID: severe and profound intellectual disability. Following Patja et al. [1]

These differences are attributed to infectious, genetic, and environmental causes [29] that have a particular impact on this subgroup of the population, especially when they have multiple disabilities. Particular genetic conditions promote both cognitive or motor disorders, as well as malignant transformations due to molecular alterations that trigger cancer [30]. In these situations, the risk of cancer overall may be increased or reduced compared to the general population, and the distribution of tumor types (tumor profile) may differ. Examples of such conditions include the conditions trisomy 13, 18, and 21 [31–33], Costello syndrome [34], Cornelia de Lange syndrome [35], Rett syndrome [36], and endocrine and metabolic pathologies [22, 37]. Some genetic conditions can also promote early-onset (e.g., childhood) cancers [30].

### Cancer risk factors in people with SPID, CP, and PMD

Risk factors for cancer are the same in people with ID as in the general population. Their impact, however, is modulated by lifestyle. The main risk factor is age; most cancers occur between the ages of 50 and 85 years. The current life expectancy of people with PMD is 55 years; therefore, many will not be affected by age-related malignancies. On the other hand, some genetic conditions that can cause PMD can also increase the risk of childhood cancer [30].

Environmental and infectious risk factors also make specific contributions to cancer incidence. In the general population, environmental risk factors are associated with an estimated 41.1% of all new cancer cases [38]. Particular risks include tobacco use (19.8%), alcohol consumption (8%), toxic occupational exposure (3.6%), and exposure to ultraviolet radiation from the sun (3.0%). Individuals with PMD tend to have a lifestyle that limits these environmental exposures. Approximately 5.4% of total cancer cases are associated with overweight and obesity. These factors, though more prevalent in the overall group of people with ID, are rare in people with PMD. On the other hand, individuals with PMD remain vulnerable to dietary risk factors for cancer (5.4%), for example due to limited fruit and vegetable intake, and possibly excess red meat and daily meat intake. This is also true for the risks related to a lack of physical activity (0.9%) and infectious factors (4%).

Infections are more common in people with ID than in the general population, especially those favored by living in institutional settings, such as *Helicobacter pylori*-related chronic gastritis, which is responsible for an excess of gastric cancer, and hepatitis B and C, which are responsible for an excess of liver cancer. Other infectious factors theoretically occur less frequently than in the general population, including HPV infection, which promotes cancers of the uterine cervix and upper aero-digestive tract [39]. Due to significant physical disabilities, the frequency of sexual intercourse tends to be lower among individuals with PMD.

Some risk factors are associated with comorbidities that occur in individuals with ID. For example, approximately 50% of individuals with SPID experience chronic gastroesophageal reflux, which strongly increases the incidence of cancer of the lower esophagus. Reflux is more frequent among those with an IQ < 35, significant motor disorders, scoliosis, or anti-convulsant medication use [40]. Similarly, neurogenic bladder conditions promote chronic lower urinary tract inflammation and bladder cancers [41]. Finally, due to the rarity of pregnancy and breastfeeding, women with PMD also have a theoretically higher risk of breast cancer compared to women in the general population [42].

### Cancer treatment and prognosis in people with PMD

The literature on cancer treatment and outcome in people with PMD is scarce. It is difficult to know how they were treated before the year 2000 [37, 43]. Women with disabilities treated for breast cancer during the period 1988–1999 in the USA had higher cancer mortality rates and were less likely to receive standard therapy than the general population [44]. This is explained by patients’ physical and psychological limitations (e.g., not able to lie flat and remain still for radiotherapy) [45, 46]. For two decades, few cases reported the indicated surgical treatment, and they did not indicate the postoperative follow-up. Only two teams provide data, including one in Italy for cancer in children with PMD. Despite many treatment modifications, sometimes excluding radiotherapy and chemotherapy, the team did not observe significant differences between the 16 children with profound ID and patients receiving normal treatment [30]. The second team in Japan treated nine adults with SPID who had cancer. Seven of them were surgically treated and alive at the time of the publication [26]. Therapeutic successes are generally reported when cancers are discovered early, such as in the tongue [23], esophagus [47], colon [24], endometrium [48], and liver [49]. A detailed review of cancer treatment and outcomes is beyond the scope of this article and deserves a focused paper. Nonetheless, the message from these two teams and isolated reports is that, diagnosed and treated early, even with many modifications, cancer can be successfully managed in people with important intellectual and physical limitations.

## Discussion

Data suggest a particular cancer distribution in persons with PMD. This profile warrants clarification by epidemiological studies on incidence, mortality, and cancer risk factors. Waiting for additional data, the current evidence can highlight risk factors in this particular population and inform the adoption of cancer screening and medical surveillance strategies to improve detection.

Taken together, a reduced life expectancy and significant reduction in risk factors could translate into a lower overall incidence of cancer during adulthood in people with PMD. This is not necessarily the case during childhood if a person carries a predisposing genetic syndrome. Yet, the impact of certain risk factors that are more common in individuals with PMD than in the general population (and those linked to genetic conditions) reduce the overall incidence gap, but without reaching the level of risk of people in the general population. New epidemiological studies are needed to precisely evaluate the incidence of cancer in people with PMD and inform targeted medical surveillance.

### A proposal for cancer screening in individuals with PMD

First, we focus on the organized screening available for the general population, then highlight some specific organ vulnerabilities.

#### Breast cancer

Breast cancer screening should be applied to women with PMD, who theoretically have an increased risk of breast cancer due to the absence of pregnancy and breastfeeding and reduced physical activity. Though publication bias is possible, the rarity of breast cancer observed in Table 2 could be due to a lower prevalence of breast cancer in Japanese woman.

Mammography screening poses difficulties related to ID. Physical challenges, particularly surrounding the ability to stand upright and raise the arm, can make examination impossible [50–53]. Alternative methods, such as ultrasound, may be used, but this method is not only operator-dependent, but more likely to yield false-positive results [50]. Future epidemiological data will make it possible to verify that breast cancer incidence justifies the disadvantages of screening as discussed for trisomy 21 [54].

#### Colorectal cancer

The risk of colorectal cancer may be increased in individuals with ID [55]. Screening is recommended, taking into account life expectancy and the fact that epidemiological data suggest a possible excess of colonic tumors. Data from Japan (Table 2) suggest that colorectal cancer could be the most frequent malignancy diagnosed in adults with PMD. The immunological test, which is easier to carry out, should encourage participation, which is still sometimes low in institutions [56]. Nonetheless, screening has allowed early discovery and treatment of colorectal cancer in persons with PMD [24, 25].

#### Uterine cervix cancer

For cervical cancer, screening is needed if there is a history or likelihood of genital intercourse because cervical cancer is mainly attributed to HPV infection [57]. Yet, this condition has not been frequently reported in women with ID. Our literature review identified only one case of cervical carcinoma, in a 15-year-old girl and unrelated to HPV infection [58] and no published case in other countries. Nevertheless, women with PMD may benefit from usual gynecological follow-up, as the uterine corpus, ovary, or other reproductive organs may present a malignant lesion (Table 2).

Unfortunately, individuals with severe disabilities have a lower, and sometimes markedly lower, screening rate for, for example, digestive tract cancer [59, 60], whereas digestive cancer is the most frequently reported in Table 2.

Concerning the benefits and harms of screening, an adapted policy should take into account two important points. The first is that age at diagnosis of cancer is lower in people with PMD than in the general population (e.g., colon cancer: mean 48.3 years vs. 72 years in the general population, esophagus: mean 39.1 years versus 71.3 in the general population; Table 2) [27]. The second point is that, for colon and breast cancer screening, the most appropriate period for people with disabilities is the one when life expectancy is > 10 years [61]. These unique features have to be kept in mind when discussing age at screening. It could be different from the general population [62].

### A proposal for medical surveillance of people with PMD

Beyond genetic conditions that confer risk for particular cancers, people with multiple disabilities may have common vulnerabilities of certain organs.

#### Esophagus and stomach

As half of the subpopulation with SPID experiences gastroesophageal reflux [40, 63], which increases the risk of lower esophageal cancer, monitoring of these individuals is necessary. Data from Japan indicate that esophageal cancer is the second most frequent cancer in persons with PMD. Symptoms can be difficult to spot, but reflux can be detected by pH-metry. Similarly, chronic gastric infection by *H. pylori* is more common in individuals with ID, especially if they entered an institution early and their stay is long [64]. Although the reduced or absent consumption of tobacco and alcohol by individuals with PMD theoretically decreases gastric cancer risk [65], chronic gastritis increases this risk and must be researched and treated to prevent this cancer.

#### Urinary bladder

Patients who experience significant urological dysfunction responsible for chronic irritation following stasis, urinary tract infections, and possible repeated urinary tract catheterization have a greater risk of urothelial cancer [66]. Urinary bladder cancer is the third most frequent malignancy in Table 2. This risk has been correlated with symptoms of the lower apparatus and the presence of spastic CP. Regular urological monitoring is desirable for these population.

#### Testes

Testes should be monitored in men with ID because the risk of testicular cancer has been estimated to be 10-times higher than in the general population [1]. Testicular cancer is an early-onset condition with a peak incidence between the ages of 15 and 35 years. Its prognosis is good if the tumor is discovered early. An important risk factor is cryptorchidism, which increases risk 3- to 5-fold. This condition can be corrected by surgical repositioning of the testicle, but treatment is decided on an individual basis [67]. Nonetheless, systematic annual monitoring is important [68]. Surveillance is easily performed by annual palpation of the testicles by a health professional of the same sex, followed by testis ultrasound if there is any concern [69].

#### Any cancer may occur

With increasing life expectancy, the incidence of cancer is likely to increase among people with PMD. As a general rule, it is necessary to consider the possibility of cancer occurring, particularly when confronted with suggestive symptoms and unexplained behavioral changes in individuals with PMD. Symptoms can be different from those presenting in individuals without ID. Furthermore, communication challenges may complicate diagnosis [70]. The importance of discovering cancers at an early stage necessitates that care providers promote screening and maintain constant vigilance. In particular, for organs at risk in individuals with PMD, performing surveillance as described above could represent an important tailored approach.

### Strengths and limitations

The strength of this review is that it was conducted by a team working on cancer in people with ID for more than 10 years and who regularly follow the literature on cancer in people with ID. This allowed us to find data and information in some articles that were not captured by the computer searches. Another strength is that we included data from Japan, where a governmental policy was directed towards people with PMD and all conference abstracts are gathered and available from the ICHUSHI source (Japanese Medical Abstracts). A weakness is that data from this source are not detailed because information is given only in abstracts without additional text. However, this allowed us to identify 135 cases, at least twice more than we found in our search of all other countries. As Japanese data are overrepresented among the published cases presented here, it is difficult to generalize the data to a global population. At a time when no review is available on the subject, this work has the advantage of being a basis for current knowledge and should encourage studies in other countries.

## Conclusion

The population with PMD was isolated recently and their cancer risk is poorly understood. Overall, the incidence of cancer among people with PMD is possibly reduced due to a limited life expectancy. Yet, some individuals with PMD have genetic conditions that place them at higher risk for certain cancers. In addition, individuals with PMD can have unique risk factors related to their living conditions and vulnerable organs. Pending consolidated epidemiological data to guide preventive and diagnostic considerations, these individuals could benefit from organized cancer screenings and surveillance of their more vulnerable organs. The importance of such a strategy is highlighted by recent therapeutic successes in children and adults that show the potential for a cure when cancer is discovered early.

### Electronic supplementary material

Below is the link to the electronic supplementary material.

Supplementary Material 1

## Data Availability

All data generated or analyzed during this study are included in this published article [and its supplementary information files].
