# Supplementary material for: Assessing cancer in people with profound and multiple disabilities
Source: BMC Cancer. 2023 Aug 25;23:798. doi: 10.1186/s12885-023-11313-3 (PMC10463777; doi:10.1186/s12885-023-11313-3)
Supplement: Supplementary file 1 — Supplementary Material 1 [file 12885_2023_11313_MOESM1_ESM.docx]

**Supplément A**

**Assessing cancer in people with profound and multiple disabilities**

Satgé et al

Arai S Horiike M, Oono K, Sahamoto H, Sumimoto S, Okuno T, Kanaoka H. a case of barrett’s adenocarcinoma in a patient with severe motor and intellectual disabilities. J Severe Motor Intell Disabil. 2015;40(2):236

*Also reported by Horiike et al 2015*

Chen WB, Matsushima A, Kawahara H, Takase S, Kosaka T. A case of Barrett’s esophageal cancer with current reflux esophagitis in a patient suffering from severe, motor and intellectual disabilities. Internal Med. 2008;101(4):804-8

Chiba Y, Kurihara M, Ohagizawa T, Eto Y. Cases of malignant neoplasms in patients with severe, motor and intellectual disabilities. No To Hattatsu. 2005;37(suppl):S392

Hashimoto H, Kodama K. Two cases of young-onset and hard-to-diagnose gastrointestinal malignancies in patients with severe motor and intellectual disabilities. J Severe Motor Intell Disabil. 2018;43(2):329

Hikino A, Takamatsu M, Shitogawa Y, Watanabe K, Mizuno Y. A case of double cancer (lung cancer and urinary tract cancer) in a patient with severe motor and intellectual disabilities. J Severe Motor Intell Disabil. 2004;21(1):91-4

Horiike M, Oono K. A case of Barrett’s adenocarcinoma with reflux esophagitis in a carry-over patient with severe motor and intellectual disabilities. Jpn J Pediatr Hematol/Oncol. 2015;52(4):338

*This report has also been done by Arai et al 2015 and by Horiike and Matsukawa 2015*

Horiike M, Matsukawa Y; A case of barrett’s adenocarcinoma after long term therapy with H2 blocker against gastroesophageal reflux disease in a patient with severe motor and intellectual disabilities. J Jpn Soc Pediatr Surg 2015;51(3):627

*This report has also been done by Arai et al 2015 and by Horiike and Oono 2015*

Idoji Y, Nakai N, Ishihama K, Yamanishi H. A case report of early detection of tongue cancer in a person with severe motor and intellectual disabilities. J Jpn Soc Disabil Oral Hlth. 2018;39(1):23-7. <https://doi.org/10.14958/jjsdh.39.23>

Inagaki T, Koide W, Nakano M, Oohashi K, Kageyama S, Ushijima K, Sugiyama S, Ogawa T, Nara K, Kaneko S, Nakano C. A patient with Rett syndrome who developed clear cell adenocarcinoma in cervix. Pediatrics of Japan. 2002;43(12):1957-9

Ishida A, Hanzawa N, Kai S, Shishikura K. A case of esophageal cancer in a patient with severe motor and intellectual disabilities. J Kanagawa Med Assoc. 2001;28(1):87

*This report has also been done by Shishikura (case 9)*

Ishido Y, Saito T, Mitsuyama K, Ishiyama A, Saito Y, Komaki H, Nakagawa E, Sugai K, Sasaki M. A case of Barrett’s esophageal cancer in a patient with severe motor and intellectual disabilities who received TS-1 for a long period. J Severe Motor Intell Disabil. 2012;37(2):313

Ishii W, Imai Y, Endo A, Arakawa C, Kohira R, Fujita Y, Fuchigami T, Mugishima H, Kodama M. Detecting brain tumor using neuroimaging in a patient with severe motor intellectual disabilities. J Jpn Soc Pediatr Radiol. 2009;25(2):126-30

Ishizaki A, Shinozaki M, Yoshida R, Kurata K, Sakamoto K, Shinohara T, Sasaki H, Sato J, Morimatsu Y. A clinical study of death in profound mental retardation with motor disturbance. No To Hattatsu. 1990;22 (4):357-63. <https://doi.org/10.11251/ojjscn1969.22.357>

Ishizuka T, Kinomiya S, Arimoto K. Terminal care in a center for severe mentally and physically disabilities. Two cases of malignant neoplasms. J Severe Motor Intell Disabil. 2003;28:57

Maruhashi K, Tsuga A, Yokoi A, Yamada S, Nakamura N, Wakisaka A, Tsuji N, Oono I. A case of advanced esophageal cancer found through lung metastases. J Severe Motor Intell Disabil. 2016;41(2):269

Matsuda M, Ookoshi Y, Hirono H, Chou H, Funahashi M, Sakurawa N, Suzuki H. Four cases of malignant neoplasms in patients with severe motor and intellectual disabilities. No To Hattsu. 2004;36(sup):S328

Matsuo K. Cancer patients factors to consider when determining treatment for breast individuals with serious mental and physical disorders. Jpn J Breast Cancer. 2019;34(3):267-9

Mizuno Y, Subiyanto K, Nakayama H. A case of Barrett’s esophageal adenocarcinoma in a patient with severe motor and intellectual disabilities. J Severe Motor Intell Disabil. 2015;40(2):235

Miyata R, Wakabayashi K, Katsura C, Kashimada K, Araki S, Ootani T, Kamiyama J. A case of esophageal adenocarcinoma with reflux esophagitis in a patient with severe motor and intellectual disabilities. J Jpn Pediatr Soc. 2007;101(2):394

Nakamura K, Kamiya Y, Ishii S, Muramatsu A, Kuroda I, Uchida N. Terminal care of patients with severe motor and intellectual disabilities having a malignant tumor. No To Hattatsu. 2017;49(3):213-4

Nishimura M, Matsumoto Y, Yagi M, Kawasaki Y. Three patients with severe motor and intellectual disabilities who developed a malignant tumor at middle age. J Severe Motor Intell Disabil. 2017;42(2):190 <https://doi.org/10.24635/jsmid.42.2_190_1>

Noda Y, Fujiwara M. Four cases of cancer in children with severe motor and intellectual disabilities receiving chemotherapy. Jpn J Pediatr Oncol. 2009;46:364

Okamura K, Oohara M, Kaneko T, Shirosaki T, Fujiwara A, Yamabuki T, Takahashi R, Komuro K, Iwashiro N, Kimura N. A case of intussusception with low-grade appendiceal mucinous neoplasm in a patient with severe motor and intellectual disabilities. Hokkaido J Surg. 2016;61(2):180

Okino F, Hirasawa K, Sato Y. Two cases of esophageal cancer with severe motor and intellectual disabilities. J Severe Motor Intell Disabil. 2003;28(2):48

Onoe S, Koda T, Nobutoki T, Watanabe M. Study of death with severely disabled people. No To Hattatsu. 2016;48(6):407-12

Origuchi Y, Myanomae T, Kogo T, Nishida T, Imai M, Sugita ***S***, Nishima S. Study of the cause of death in patients with severe motor and intellectual disabilities syndrome. Medical background in the dead cases. Jpn J National Med Services. 2001;55(4):175-9

*Also reported by Origuchi and Myanomae 2002*

Origuchi Y, Myanomae T. Causes-of-death analysis based on age at death in patients with severe motor and intellectual disabilities syndrome. Jpn J National Med Services. 2002;56(8):476-8

*This report has also been done by Origuchi et al 2001*

Osawa S, Oosaka Y, Kawamitsu T, Hamada A, Tachibana A, Deguchi M, Sugimori J. Two cases of malignant tumors in patients with severe motor and intellectual disabilities in our hospital. Jpn J National Med Services. 2001;55(suppl1):130

Sado T, Nishimura T, Fujii H, Ueda M, Takayama K, Kurata K, Matsuzaki S, Kitahara M, Iwasaki Y. Patients with severe motor and intellectual disabilities who developed malignant neoplasms during a long period of admission. J Nagano Med Assoc. 2020;50:94-5

Saito N, Kakihaha T, Araya T. Palliative care to a patient with severe motor and intellectual disabilities who developed a malignant tumor (first report). J Severe Motor Intell Disabil. 2017;42(2)242 <https://doi.org/10.24635/jsmid.42.2_242_2>

Sano K, Ayashida S, Ito M, Ogasawara K, Fujiwara M. A case of hepatoblastoma in a patient with severe motor and intellectual disabilities. J Jpn Soc Pediatr Surg. 2008;44(1):80-1

Shibaki T, Ikegami J, Akabane H, Nakano S, Inagaki M. A case of a patient with severe motor and intellectual disabilities who is given trastuzumab alone against metastatic breast cancer. Hokkaido J Surg. 2015;60(2):222

*Also reported by Shibaki 2016*

Shibaki T, Iekue S, Akabane H, Yanagida N, Inagaki M, Nakano **S**. A case of a patient with severe motor and intellectual disabilities who underwent treatment of metastatic breast cancer with trastuzumab alone. J Jpn Surg Assoc. 2016;77(9):2170-4 <https://doi.org/10.3919/jjsa.77.2170>

Shibuya I, Motoki T, Ueda R, Saijyo H, Shimizu-Motohashi Y, Nakagawa E, Sugai K, Sasaki M. Bladder cancer in individuals with severe motor and intellectual disabilities: A report of two cases. J Severe Motor Intell Disabil. 2016;41(3)439-44 <https://doi.org/10.24635/jsmid.41.3_439>

Shishikura K. Carry over : practical experience of persons with severe motor and intellectual disabilities. No To Hattatsu. 2005;37:225-30

*Also reported by Ishida et al 2001*

Sugimori M, Meijou K, Ito T. Three cases of colon cancer in patients with severe motor and intellectual disabilities. J Severe Motor Intell Disabil. 2016;41(2):247

Taira Y, Kumagaya K, Katayama M, Hanada K, Takeuchi E, Asano T, Inoue H, Takeuchi A, Oka M, Ootsuka S. Metastatic multiple bone tumors from thyroid cancer in a patient with severe motor and intellectual disabilities. J Severe Motor Intell Disabil. 2011;36(2):280

Takeshima T, Sakata R, Kobayashi M, Yokomizo Y, Oosaka K, Takano T, Fujikawa A, Oouchi H, Tsuchiya F, Iwasaki H. Two cases of testicular cancer developing within cryptorchidism in patients with severe motor and intellectual disabilities. J Kanagawa Med Assoc. 2011;38(1):84

Takei K, Miura T, Hosoda M. A case of gastro-cardiac cancer in a patient with severe motor and intellectual disabilities. J Severe Motor Intell Disabil. 2004;29(2):185

Takeushi T, Morohoshi T, Chiba S. A case of endometrial cancer treated with conservative therapy. J Severe Motor Intell Disabil. 2005;30(2):217

Tokumistu A, Yamamoto M, Kusuoki Y, Hiramoto A. Malignant neoplasms in patients with severe motor and intellectual disabilities hospitalized in Hokkaido Ryoikuen. No To Hattatsu. 2004;36(suppl);S227

Tokunaga O. The transition and the current situation of medical care for patients with severe motor and intellectual disabilities in our hospital. Jpn J National Med Services. 2018;72(5):213-8

Tominaga K, Omine S, Ito M. Four cases of tumor-like lesions in patients with severe motor and intellectual disabilities. J Severe Motor Intell Disabil. 2008;33(2):254 (b09)

Tsujiyama A, Odaka T, Kanamori Y, Tanaka Y, Terawaki K, Furumura M, Sugiyama M, Odajima S, Ito J, Iwasaki H, Fukuda S, Iwanaka T. A case of Barrett’s esophageal cancer diagnosed in a patient aged 43 years suffering from severe and motor intellectual disabilities. J Jpn Soc Pediatr Surg. 2011;47(6):974

Yamada N, Shikura K. A case of multiple myeloma and mixed connective tissue disease in a patient with severe, motor and intellectual disabilities. J Severe Motor Intell Disabil. 2008;33(2):254 (b10)

Yasuda K. Three cases of tumor in/around the liver in patients with severe motor and intellectual disabilities. Annual meeting of the Japanese Society of National Medical Services. 2014;68:459
